# Haiti’s Commitment to Malaria Elimination: Progress in the Face of Challenges, 2010–2016

**DOI:** 10.4269/ajtmh.16-0902

**Published:** 2017-10-18

**Authors:** Jean Frantz Lemoine, Jacques Boncy, Scott Filler, S. Patrick Kachur, David Fitter, Michelle A. Chang

**Affiliations:** 1 Programme National de la Contrôle de la Malaria, Ministère de la Santé Publique et de la Population, Port-au-Prince, Haiti;; 2 Laboratoire National de la Santé Publique, Ministère de la Santé Publique et de la Population, Port-au-Prince, Haiti;; 3 The Global Fund, Geneva, Switzerland;; 4 Division of Global Health Protection, Centers for Disease Control and Prevention, Port-au-Prince, Haiti;; 5 Division of Parasitic Diseases and Malaria, Centers for Disease Control and Prevention, Atlanta, Georgia

## Abstract

Haiti is committed to malaria elimination by 2020. Following a 2010 earthquake and cholera epidemic, Haiti capitalized on investments in its health system to refocus on malaria elimination. Efforts, including expanding diagnostics, ensuring efficacy of standard treatments, building institutional capacity, and strengthening surveillance were undertaken to complement the broad health system strengthening activities. These efforts led to the adoption and scale-up of malaria rapid diagnostic tests as a diagnostic modality. In addition, drug-resistant monitoring has been established in the country, along with the development of molecular testing capacity for the *Plasmodium falciparum* parasite at the National Public Health Laboratory. The development and piloting of surveillance activities to include an enhanced community-based approach for testing and treatment of patients has increased the ability of the Ministry of Health to map foci of transmission and respond promptly to outbreaks. The reinforcement of evidence-based approaches coupled with strong collaboration among the Ministry of Health and partners has demonstrated that malaria elimination by 2020 is a realistic prospect.

## INTRODUCTION

Following a devastating earthquake in Haiti in January 2010, there was robust interest in rebuilding the health infrastructure in the country.^1,2^ Donors stepped forward motivated by a renewed vision for investing in the country’s programs that would not only mitigate the acute damage left by the natural disaster, but improve the health infrastructure in a meaningful, long-term manner. The progress made by the Program National de la Contrôle de la Malaria (National Malaria Control Program in French; PNCM), with international assistance, is a good example of how to rebuild and advance in a crisis setting.

The PNCM in Haiti, like many other countries that participated in the Global Malaria Eradication Program, saw a drop in financial resources after the program ended in 1969.^3^ In 1988, a major financial crisis in Haiti ended its domestic elimination program, which left the national malaria program significantly underfunded.^4^ During the same year, the country reported 12,000 cases of malaria.^5^ In the following years, Haiti’s national program did not receive substantial external funding until The Global Fund (GF) was launched. The first GF grant for malaria in Haiti was awarded in round 3 ($12.8 million) implemented during 2004–2009, the years before the earthquake.^6^ The main areas of focus for the program were service delivery, supporting the departmental infrastructure for case management including microscopy testing for malaria. Other investments were made in routine reporting, as well as the sale and free distribution of long-lasting insecticidal nets (LLINs). Notably, collaboration for planning malaria control efforts with the Dominican Republic (DR) was an objective within this first grant. Although progress in malaria control was made throughout the 2000s, it was encumbered by the changing political climate including a political coup in 2004, followed by a 2-year period of stalled presidential elections and heightened insecurity, which was disruptive to implementation of activities. Given this context at the time, the program’s goal was still malaria control, until elimination again became a prospect.

The progress made by Haiti and the DR through these early years of working together resulted in a 2009 binational plan for malaria elimination by the year 2020.^7^ The plan complemented the commitment made earlier by both countries to support the Millennium Development Goals.^8^ The cross-border efforts of the ministries, technical partners, and donors fostered harmonizing malaria treatment policies, technical practices for diagnostics, and sharing surveillance data. Although this plan developed by Haiti and the DR did not attract the financial support to make it viable, the collaboration between the ministries and partners helped shape the next GF grant application that included cross-border collaboration with the overarching objective of malaria elimination on Hispaniola.

The emerging political commitment for malaria elimination and the prospect of a larger GF grant signified advancement; however, the 2010 earthquake tragically killed an estimated 220,000 people in Haiti and disrupted the prospect of rapid progress for any health program.^9^ In the aftermath of the earthquake, with the extent of investments in health systems there was an opportunity to engage more carefully and to invest in several aspects of the malaria program to approach elimination more effectively.

## DIAGNOSTICS—ESSENTIAL FOR INFECTIOUS DISEASES SURVEILLANCE AND MALARIA ELIMINATION

The humanitarian organizations that arrived in Haiti following the earthquake had a remit to provide emergency medical care for the injured and displaced population. Estimates of the number of nongovernmental organizations (NGOs) providing humanitarian or development assistance in Haiti before the earthquake range widely from 3,000 to 10,000.^10^ The multitude of NGOs arriving in Haiti contributed to the establishment of temporary clinics and camps. The additional organizations also introduced more complexity by multiplying the coordination challenges and introducing new partners who were not experienced in Haiti.^11^ The various health-care sites by the responding partners needed to be incorporated into the surveillance system, as concern for infectious disease outbreaks due to population displacement, and disruption of essential services following a disaster, is high.^12,13^ Though testing for malaria was a necessary part of the diagnostic algorithm for fever, testing could not be supported by traditional parasitological microscopy because of the high burden of fever in this setting, coupled with poor infrastructure. Some response organizations brought malaria rapid diagnostic tests (RDTs), which were not accepted as standard policy for use in Haiti at that time. First-generation RDTs had performed inadequately for malaria diagnosis in Haiti earlier; thus, the national program maintained microscopy as the diagnostic standard.^14^ Nevertheless, in the context of the emergency and the need for confirmatory testing under difficult conditions, expanding microscopy was not optimal.

**Figure 1.**
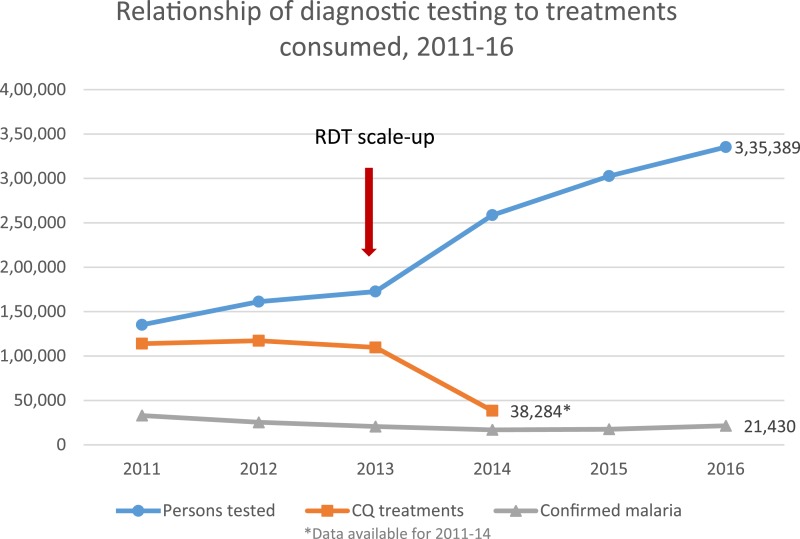
Relationship of diagnostic testing to treatments consumed, 2011–2016. This figure appears in color at www.ajtmh.org.

The Ministère de la Santé Publique et de la Population (Ministry of Public Health and Population in French; MSPP) decision to allow RDTs to be used for a temporary 3-month period for the humanitarian response served an immediate need, but the 2020 goal of malaria elimination would be more difficult to support if microscopy were the only option beyond the acute response phase. The vision to establish a permanent policy for the use of RDTs in Haiti would satisfy the needs of the emergency response, and establish momentum for the rapid scale-up of malaria diagnostics for routine care. In addition, the risk that responding NGOs might bring RDTs that were not fully evaluated by the global product testing program could be minimized because the MSPP could enforce standards defined in a national policy.^15^ The MSPP and Centers for Disease Control and Prevention (CDC) worked to rapidly conduct a field trial of the RDTs that were in use in Haiti, assessed their performance compared with microscopy, and used this information, along with the global product testing results, to develop a permanent national policy for use of RDTs in malaria diagnosis.^16^ The established national policy was a critical prerequisite for reprogramming GF funds for RDT procurement; the GF has supported procurement of RDTs since 2013, when phase 2 of the GF grant was revised to include the implementation of RDTs. The incorporation of RDTs into Haiti’s national policy has led to the ability to test for and confirm malaria illness at all health facilities. The roll out of this more easily useable diagnostic test was associated with notable decreases in presumptive treatment and the use of chloroquine. Currently, the malaria confirmation that is reported in the national surveillance is done predominantly by RDT compared with microscopy at a ratio of greater than 3:1, and with RDTs predominantly found in the mid- to low-level health facilities. These lower level health facilities more commonly serve communities in rural areas where malaria transmission is higher. The impact of the adoption of the national policy and scale-up of RDTs can be seen by the increase in the number of completed malaria tests from 135,136 to 302,740 in 2011 and 2015, and the associated decrease in the number of treatments consumed more aligned with the number of confirmed cases (Figure 1).

## ENSURING EFFICACY OF ANTIMALARIAL DRUGS AND PREVENTIVE INTERVENTIONS—NOW AND IN THE FUTURE

Chloroquine is the first-line treatment of *Plasmodium falciparum* malaria in Haiti. The drug remains sufficiently efficacious against the parasite in only a few other places in the world.^17^ The malaria-endemic countries in the Caribbean and Central America are fortunate in the respect that they are still able to use an inexpensive, safe drug for treatment, as opposed to more expensive artemisinin combination therapies required in all other parts of the world. Routine monitoring for the continued efficacy of a country’s first- and second-line antimalarial drugs should be done as part of all endemic countries’ national programs.^17^ The last therapeutic efficacy studies available at the time of the earthquake had been conducted in Haiti in the 1980s, and only a single study from one area of the country conducted in 2006 reported testing parasites for molecular markers of chloroquine resistance.^18,19^ As part of the initial response to the 2010 earthquake and to assess rapidly if chloroquine resistance was a problem, MSPP and CDC collected blood samples to evaluate the presence and frequency of validated molecular markers for chloroquine resistance.^20^ In addition, they collaborated with other research partners to examine previously collected samples to aid in assessing the status of chloroquine resistance. The two studies by Charles and others and Morton and others together contributed results from 1,009 samples that were collected over the consecutive years from 2005 to 2010 from multiple sites.^21,22^ The analysis for mutations in the *pfcrt* gene found three of 1,009 (0.3%) samples with chloroquine-resistant haplotypes, of which two could not be unequivocally verified. Additional studies on the population genetics of the parasites revealed that the *P. falciparum* clones in Haiti are highly related and distinct from other countries. The genetic analysis suggested that the parasites with chloroquine-resistant haplotypes were possibly imported, but are not circulating at any sustained or significant level in Haiti. These results reassured the program, partners and policy makers that chloroquine was still an efficacious drug in Haiti.

Although two therapeutic efficacy studies (MSPP unpublished report) were completed in 2015 and confirmed the continuing sensitivity of the parasite to chloroquine, these were the most recent after several decades without an in vivo study.^23^ The logistical challenges experienced in the recruitment and follow-up of patients are an obstacle to conducting them routinely. The infrequency of therapeutic efficacy studies, completed between the 1980s and the present, argues for the surveillance of the emergence of drug resistance by monitoring molecular markers for resistance to chloroquine. Drug-resistant monitoring is now an established part of the program in Haiti; 11 sentinel sites collect samples for antimalarial drug-resistant testing by molecular methods. To date, the drug-resistant monitoring program has collected 919 samples during 2016. Of these, 668 have been sequenced and no molecular markers for chloroquine resistance have been detected (MSPP/CDC unpublished data, 2017).

An additional issue that has arisen is the deletion of the *histidine-rich protein 2* (HRP2) gene in *P. falciparum*, which interferes with the ability of the commonly used RDTs to detect infection. This has been identified and documented in several countries in South America; however, parasites carrying the mutation for HRP2 deletion have not been identified in countries west of the Panama Canal.^24,25^ To ensure the efficacy of the main diagnostic tool for malaria in Haiti, these 11 sites will continue to monitor for both molecular markers of antimalarial drug resistance and begin to monitor for parasite mutations that would interfere with the HRP2-based RDTs.

Ensuring that the strategies are effective is critical to reaching the goal of malaria elimination by 2020. Resources that are spent on interventions that have a modest or no impact compromise the optimal use scarce resources, further widening the gap between the need and that which is secured. Mass distribution of LLINs was a primary intervention funded in the round 8 GF grant for years 2011–2015. The budget for the procurement and distribution of LLINs was approximately two-thirds of the GF grant, which left little remaining budget for investing in improving diagnostics or surveillance. Unlike the primary vectors in sub-Saharan Africa where LLINs have been well-documented to significantly decrease malaria transmission, disease burden, and child mortality,^26–28^ the malaria vector in Haiti is *Anopheles albimanus* which tends to bite outdoors and at variable times throughout the evening and nighttime hours—situations that are not typically protected by bednet use.^29–32^ The results from studies assessing the impact of LLINs in countries in the region of the Americas that have the same vector are mixed.^33–36^

The opportunity to evaluate the effectiveness of LLINs in Haiti was an important step toward refining the evidence base for tools that could be used for malaria control and elimination for Haiti. Approximately, 3 million permethrin-treated nets were distributed in Haiti in 2012 during a nationwide, mass campaign. This was followed by a case–control study to assess the effectiveness of the LLINs in the Haitian context with a primary vector that has variable biting habits. The study suggested that the mass LLINs had no significant effect in preventing clinical malaria (odds ratio = 0.95, 95% confidence interval = 0.68, 1.32).^37^ Although the study design could not rule out a modest impact that was not measurable, these findings are compatible with vector behavior studies in Haiti from the published literature,^32^ as well as from recent evaluations conducted by the PNCM and CDC, that show predominantly outdoor biting but some indoor biting during early evening hours (Impoinvil D, personal communication). After this experience, the PNCM targeted LLIN distribution to the medium- and high-transmission strata of the country.^38^ Implications from the mass distribution and study results suggest that a strategy for malaria control/elimination in Haiti might be better served with alternate interventions that target the parasite reservoir, or if proven effective, with novel vector-control strategies such as attractive toxic sugar baits or spatial repellents, that might address earlier biting times and outdoor biting of mosquitoes.

## BUILDING CAPACITY AT HAITI’S NATIONAL PUBLIC HEALTH LABORATORY

An insectary was established and entomological technicians were trained at the Laboratoire National de Santé Publique (National Public Health Laboratory in French; LNSP) to fill a gap highlighted by the earthquake, while keeping the vision of building infrastructure to maintain progress toward elimination. With displaced populations living in makeshift shelters, the possibility of using insecticides to reduce the mosquito vector population depended on knowing the insecticide-resistant profile in the country. The capacity to test and monitor for the development of mosquito resistance to insecticides, and to assess the behavior of the mosquito vector (to tailor interventions), was established in Haiti. The insectary enables the MSPP to conduct insecticide-resistant testing and to rear mosquitoes necessary for other vector-control assessments such as testing the insecticidal activity of LLINs. In addition, the entomological technicians provide technical support to a larger network of vector-control agents whose duties involve identifying and characterizing larval development sites and applying larvicides to these sites for control. The technical capacity of the insectary continues to expand, and is now being leveraged to assist in other urgent health outbreaks due to dengue, chikungunya, and Zika viruses.

Strengthening of molecular testing capacity at the LNSP will assist in the long-term goal of malaria elimination. National parasite prevalence surveys were conducted in 2011, 2012, and 2015 as part of Haiti’s GF grant, round 8. These were the first national level household surveys to be done in Haiti for *P. falciparum* parasitemia. A growing body of knowledge suggesting that asymptomatic parasitemia (usually low density infections) could be a substantial proportion of infections in low-transmission settings contributing to sustaining transmission^39^ prompted the MSPP to include polymerase chain reaction (PCR) testing, along with microscopy and RDTs in the first survey. The results of the 2011 survey documented that Haiti’s national *P. falciparum* prevalence was very low, approximately 0.4% by PCR.^40^ The results for the subsequent surveys in 2012 and 2015 were consistent with this first parasite prevalence measurement. PCR capacity at the LNSP for *P. falciparum* was developed to serve the needs of the country to be able to test for low-density infections that could be missed by other testing modalities. Molecular testing could also support future malaria elimination strategies that include identifying low-density infections.

## STRENGTHENING AND EXPANDING SURVEILLANCE TO IDENTIFY MALARIA FOCI

To more thoroughly identify malaria infections, map the locations where infections occur, and to rapidly test and treat, the MSPP implemented a community-based surveillance system in the commune of Ouanaminthe in the Northeast Department of Haiti, bordering the DR. This was the site of a pilot that supported community testing and collaboration between the two countries. Field agents responsible for searching for people with recent fever circulate through the commune carrying RDTs and chloroquine plus single-dose primaquine, the first-line treatment, to provide this service. This pilot system detected early an increase in malaria cases in 2013 in one section of the commune. Based on this early detection, the MSPP was able to respond promptly with mass testing and treating in the community and larviciding of breeding sties, and stopped the outbreak—cases decreased from the peak of 42 in June to three during the following month (PNCM/MSPP, surveillance data 2013). The surveillance system provides information to map where cases occur in an attempt to identify the important geographic areas where transmission is the most problematic. The mapping of transmission foci allows the program to target interventions, whether vector control or another intervention that could accelerate the progress to malaria elimination.

Another pilot spearheaded by the PNCM to begin mapping high-transmission foci using passively identified malaria cases was implemented in Grand Anse, Sud, and Sud-Est Departments of Haiti. In areas where there are relatively higher levels of transmission, the cases identified at the health facilities may offer valuable information as to where most infections are occurring. The challenge in many countries where there is no system of addresses to verbally collect where someone resides poses a challenge to mapping transmission through the health facilities. Using field agents trained in reviewing health facility records, locating the patients at their residence, taking a GPS coordinate, and completing a short questionnaire allows the PNCM to begin identifying areas of higher transmission through the routine health system. This pilot will be valuable for understanding the feasibility of localizing malaria case clusters using passive surveillance data and for identifying transmission foci. Additional lessons learned from this experience include understanding what information is the most useful for locating residences to follow-up cases and understanding the quality of current facility records.

Finding opportunities to piggy-back testing for malaria exposure (serological presence of antibodies to malaria antigens) on to other disease surveys has been another approach to gather information to localize transmission foci. One prime example of collaboration between different disease programs is the incorporation of malaria testing into the national lymphatic filariasis (LF) transmission assessment surveys (TAS). The LF TAS, a standard component of LF elimination programs, is typically school based, if the net primary-school enrolment ratio is ≥ 75%. The survey is designed to provide a “pass-fail” benchmark for areas that could be considered free of LF transmission after implementing mass drug campaigns for LF treatment. Although the ideal sampling strategies for identifying malaria foci compared with LF-free zones differ, integrating testing is an opportunity to obtain additional data points that can contribute to the mapping of malaria transmission foci without significant additional cost. Since 2014, the PNCM has been able to take advantage of the ongoing LF TAS in 21 of 23 total evaluation areas in the country to obtain information on malaria transmission by conducting an integrated TAS.^41^ Preliminary results from one evaluation area, the Department of Nippes, that incorporated malaria testing into the LF TAS, found zero children who were positive by RDT. However, serology results from the same children showed that those in 34 of the 43 schools had evidence for previous exposure to malaria by the presence of antibodies against *P. falciparum* (PNCM data/Rogier E, personal communication). These results highlight the advantage of serologic testing compared with the use of RDTs in low-transmission settings. Ultimately, the incorporation of malaria serology into the LF TAS will contribute to localizing the foci of high malaria transmission in Haiti.

## CLOSER TO MALARIA ELIMINATION

**Figure 2.**
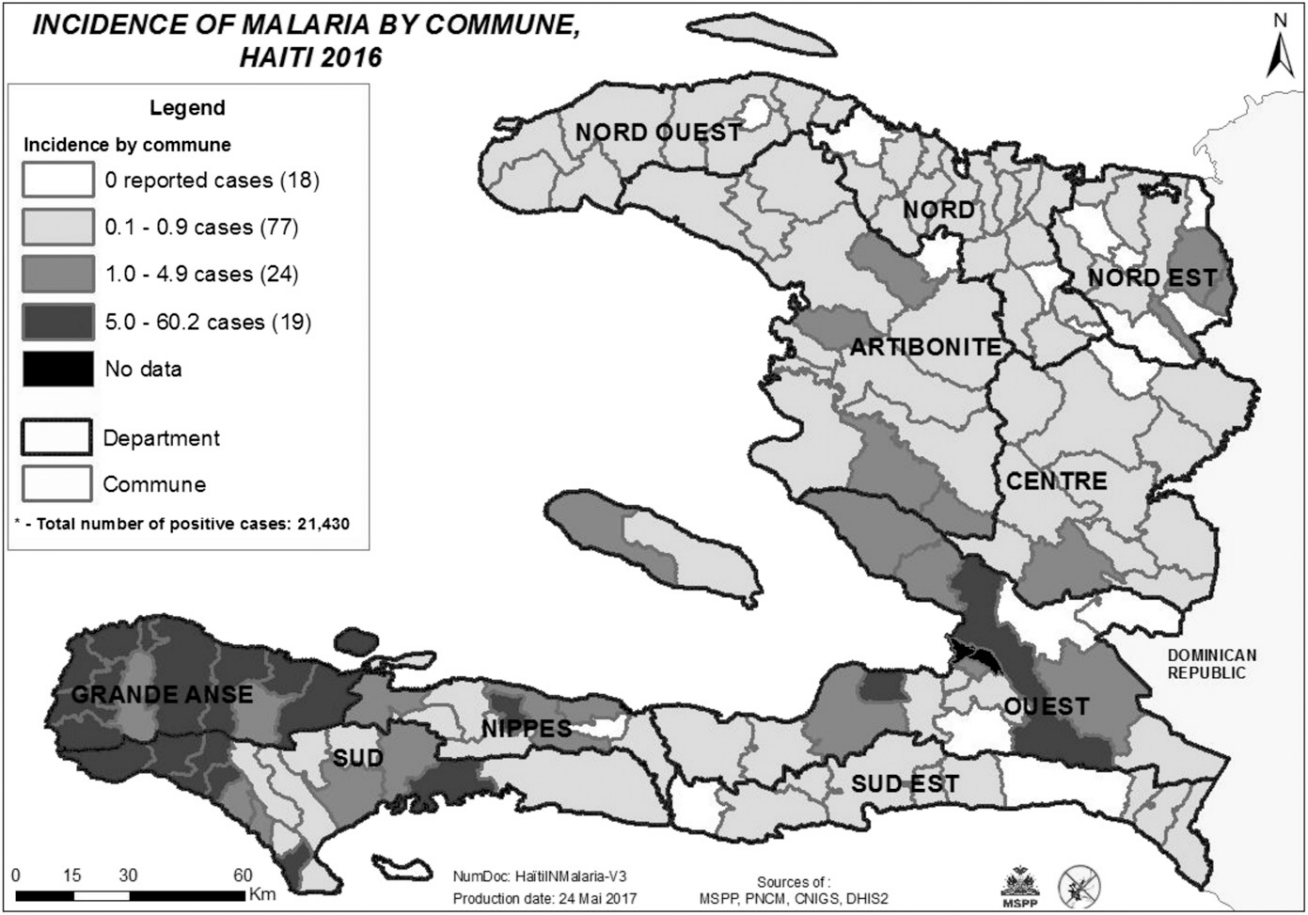
Communes stratified by malaria incidence per 1,000 populations, 2016.

The 6 years that followed the earthquake in Haiti presented an opportunity to start anew and rebuild components of the public health infrastructure. It was also a period of risk for ongoing programs and initiatives with longer term goals which could be compromised because of the importance and momentum to focus on the immediate needs related to the natural disaster, or the related and other disease outbreaks (i.e., cholera, chikungunya, Zika) in the subsequent years. The national malaria program was able to use the existing and new resources intended for postearthquake reconstruction to support the emergency response and recovery, while also accelerating the country’s momentum toward malaria elimination. The program succeeded in developing a foundation for malaria elimination by strengthening its evidence base and building capacity in critical areas such as surveillance and diagnostics, resulting in the ability to stratify and prioritize communes for interventions (Figure 2). The additional strategic factors that allowed the PNCM to succeed in maintaining progress included keeping sight on the long-term vision of elimination and building on each step of progress despite recurrent interruptions; fostering the coordination of activities and partners—whose numbers can wax and wane depending on funding opportunities; and finally, integrating with other disease programs when possible and advantageous. The time remaining until 2020, the year to reach malaria elimination, is short, with much left to accomplish. However, the progress made during the years following the 2010 earthquake demonstrates that with additional financial resources, partner support, coordination, and leadership, the goal of malaria elimination is an achievable one.

### Addendum.

Hurricane Matthew devastated areas of Haiti in October 2016, making landfall in the southern departments that report the most malaria cases in the country. The disaster response efforts will contribute to rebuilding malaria service delivery and surveillance systems for the immediate posthurricane needs, while continuing to strengthen the systems that are essential for malaria elimination. Hurricane Matthew was another reminder of the importance of maintaining the long-term vision of malaria elimination, while adapting to the inevitable challenges along the way.
